# Two-Dimensional MXenes for a Sustainable Future

**DOI:** 10.1021/jacs.6c11786

**Published:** 2026-08-28

**Authors:** Guilherme Ribeiro Portugal, Yury Gogotsi, Johanna Rosen

**Affiliations:** † Materials Design, Department of Physics, Chemistry and Biology (IFM), 4566 Linköping University, SE-581 83 Linköping, Sweden; ‡ Wallenberg Initiative Materials Science for Sustainability (WISE), Linköping University, SE-581 83 Linköping, Sweden; § A. J. Drexel Nanomaterials Institute and Department of Materials Science and Engineering, Drexel University, Philadelphia, Pennsylvania 19104, United States

## Abstract

Two-dimensional (2D) carbides, nitrides, and related compounds known as MXenes are the fastest-growing family of inorganic nanomaterials, which promise to advance communication, energy, water, and healthcare due to their unique and tunable properties. To deliver on this promise, one needs to scale up their manufacturing in a sustainable way and evaluate their capacity to enhance the efficiency of thermal management and solar energy harvesting, storing renewable energy, desalinating water, and reshape our lives through the introduction of new wearable devices. This Perspective provides a critical analysis of green and sustainable MXene manufacturing and an overview of MXene applications in sustainable technologies, such as energy generation and storage, thermal management, sensing, electromagnetic interference shielding, and water desalination and remediation. The most promising material solutions are suggested, including hybridization with other materials with complementing properties to further enhance MXene performance and build devices and systems that cannot be created using conventional materials. Computationally guided waste-free assembly of MXenes with other nanoparticles and organic molecules can accelerate discovery and reduce trial-and-error in building next-generation systems, opening pathways to sustainable technologies that will shape our future.

## Graphical abstract

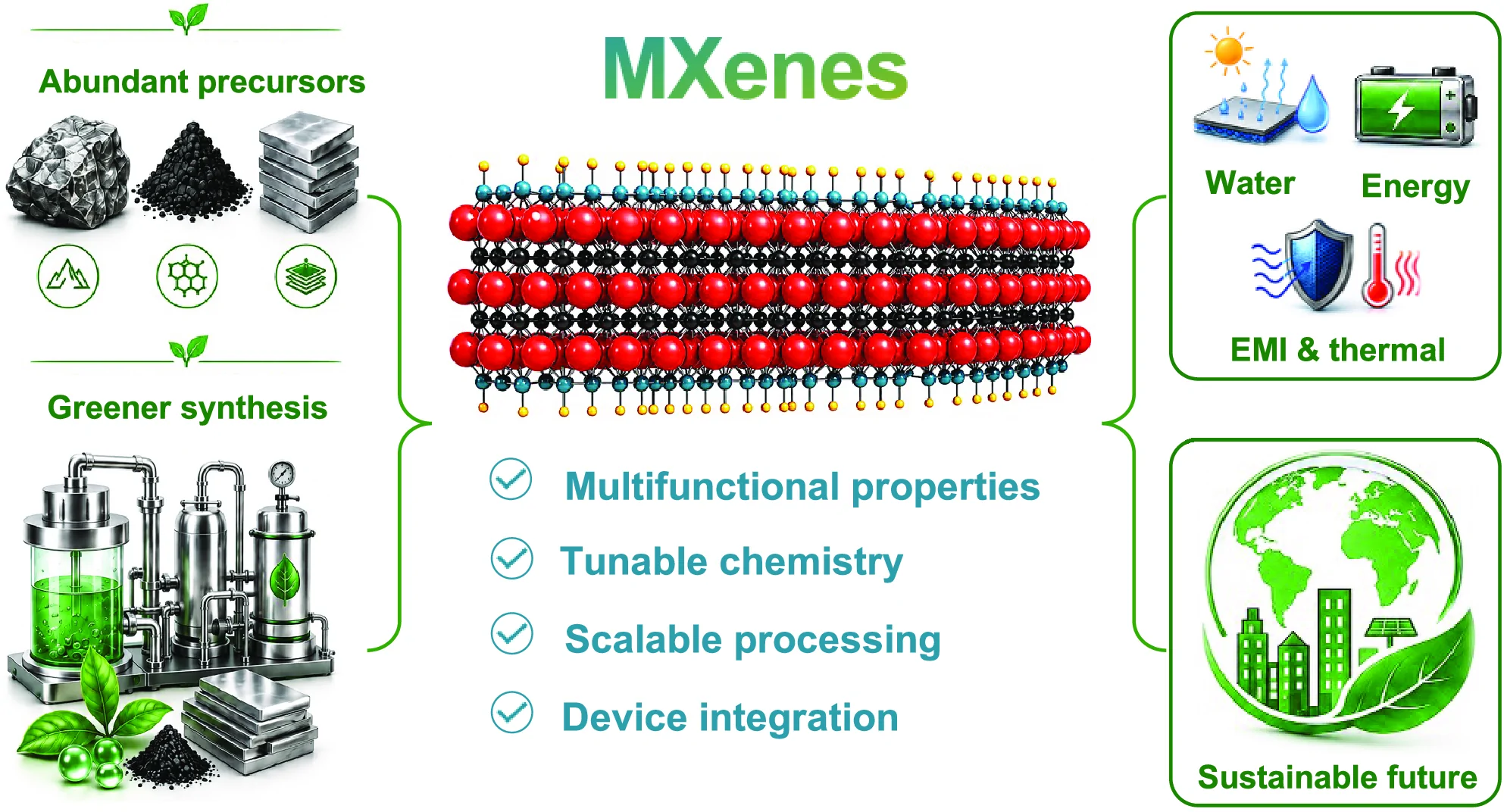

The latest chemistry Nobel Prizes for metal–organic frameworks (2025) and quantum dots (2023) have once again underscored a well-known paradigm: mastery and development at the nanoscale are now key to unlocking a new technological era. In fact, the progression of human society has been defined by the design and processing of new materials throughout history, from the smelting of bronze and iron to the forging of steel and the doping of silicon. Likewise, nanoscience may well be responsible for launching a new epoch in which chemically assembled materials leverage step-changes in performance and integration, especially in the sustainability context, where nanoscale building blocks enable waste-free device manufacturing and reshape how we harvest, store, and convert energy.^1–5^ Nowhere is this more evident than in the progress of two-dimensional (2D) crystals, which, since the isolation of graphene,^6,7^ have evolved from a scientific curiosity into a central platform for condensed-matter physics and materials science.^8–10^

Among the promising 2D materials for a sustainable future, we are witnessing the rise of MXenes, a versatile class of transition-metal carbides, nitrides, and carbonitrides whose inherently tunable chemistry and surface properties offer a unique design space for high performance and environmentally conscious processability.^11–14^ Since the first MXene report in 2011,^15^ the annual number of MXene-related publications has grown significantly and keeps increasing faster than for other 2D materials. Their generic formula, M_*n*+1_X_*n*_T_*x*_ (*n* = 1–4), typically comprises early transition metals (M) bonded to C and/or N (X), with variable surface terminations T_*x*_ (such as O, OH, halogens, chalcogens, etc.) whose composition and stoichiometry (*x*) are set by the synthesis protocol and post-treatment.^16–18^ As the inaugural structure,^15^ Ti_3_C_2_T_*x*_ is by far the most investigated MXene, typically obtained by selectively etching the Ti_3_AlC_2_ MAX precursor phase in aqueous hydrofluoric acid (HF). Rather than being a single compound, MXenes constitute a whole design space, with approximately 60 distinct experimentally realized stoichiometric compositions, dozens of solid solutions and high-entropy materials, and over a thousand predicted stoichiometric variants when surface terminations are considered.^19,20^ Recently, several lanthanide-based semiconducting MXenes were claimed,^21^ and predictions suggest that other lanthanides and actinides could also form MXenes.^19^

Recent advances in synthesis and theoretical modeling enable the control of MXene structures and chemistries at (near) atomic precision across this large design space. Researchers have achieved accurate control over fundamental structural parameters, including the number of atomic layers,^16^ atomic ordering (both in-plane^22^ and out-of-plane^23^ ordered phases and a combination thereof^24^), the formation of M/X-site solid solutions,^16^ and high-entropy systems incorporating up to nine different metals at the M-site (see Figure 1).^25–27^ Importantly, in Figure 1, the superordered *s-*MXene is shown schematically for *n* = 5, although experimental realization has thus far been reported for *n* = 4,^24^ reflecting the rapid expansion of MXene structural diversity. Such a level of chemical manipulation allows for on-demand tuning of properties, providing MXenes with a customizable palette of ionic, electronic, and surface properties that can be engineered for specific target applications.^28–30^ Together, these attributes make MXenes promising candidates for large-scale sustainable applications. Systems based on Earth-abundant elements (e.g., Ti, Nb, Mo, C, N) have exhibited high performance in electrochemical energy storage,^31,32^ efficient light-to-heat conversion and thermal management,^33,34^ water and air remediation,^35–37^ among several others. Moreover, the synthesis toolbox has expanded beyond the initial methods to include aqueous topochemical routes,^38^ supercritical CO_2_ as a solvent,^39^ Lewis-acid molten-salt (LAMS) etching,^40,41^ selective etching in halogen-containing gases,^39,40^ gas-phase synthesis and chemical vapor deposition (CVD),^44–46^ as well as other methods that offer distinct pathways to optimize cost, throughput, and environmental impact. The successful scaling of archetypal MXenes like Ti_3_C_2_T_*x*_ ^47^ and Mo_2_CT_*x*_ ^48^ from grams to industrially relevant quantities shows a realistic route toward meeting market needs for large-scale manufacturing.

**Figure 1:**
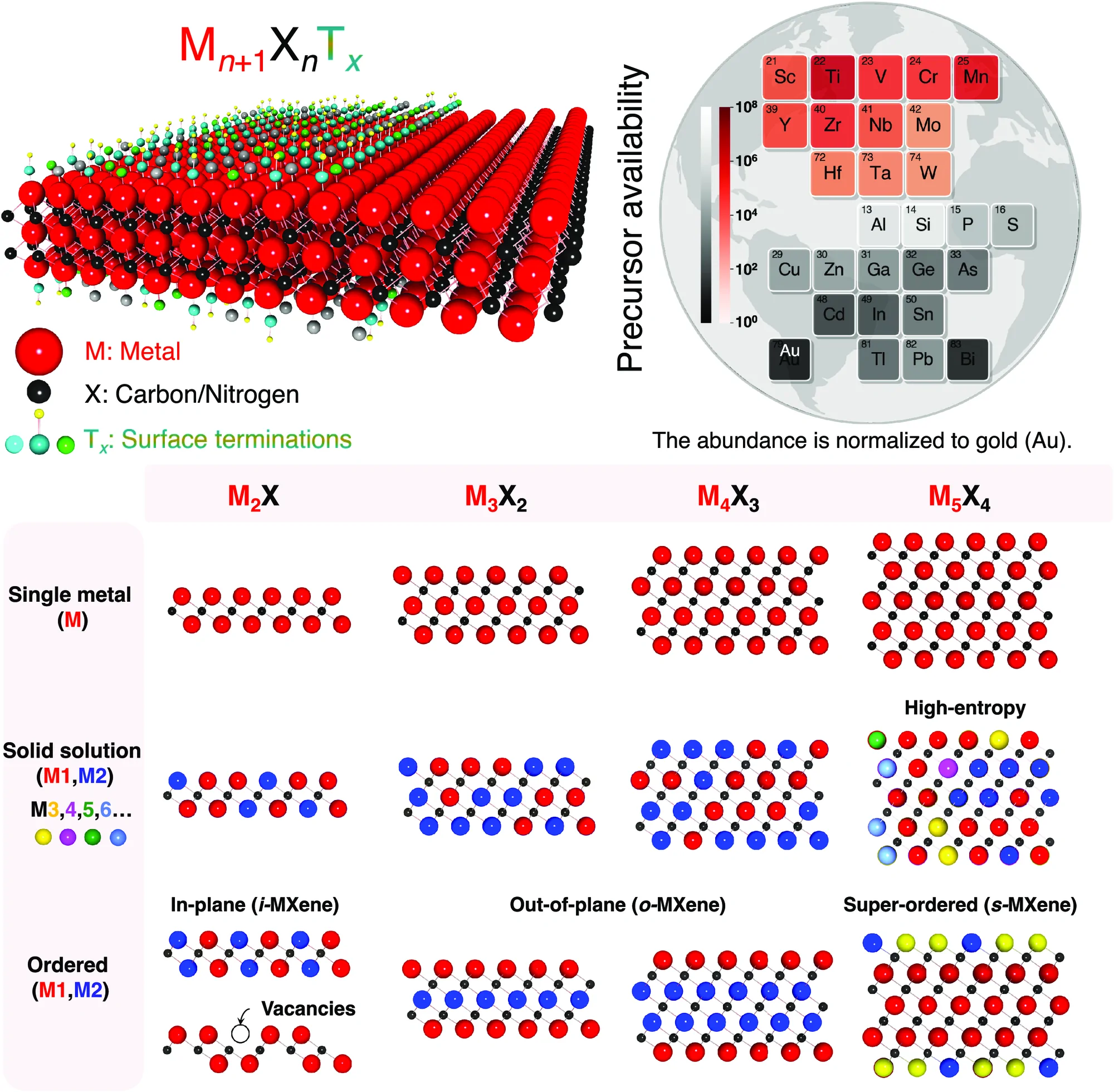
MXene structural diversity and precursor availability. A representative MXene lattice (M_*n*+1_X*_n_* T_*x*_) highlights the metal–carbon/nitrogen framework and surface terminations, with M shown in red, X = C/N in black, and *T*_*x*_ terminations in cyan/yellow/gray. The accessible structural landscape is then summarized across increasing MXene thickness (M_2_X to M_5_X_4_), spanning single-metal sheets, solid-solution compositions (M1, M2 and multicomponent mixtures, including high-entropy MXenes), and ordered variants featuring in-plane metal/vacancy ordering (*i*-MXene), out-of-plane ordering (*o*-MXene), and superordered (*s*-MXene) structures. Precursor availability for representative elements relevant to MXene synthesis is contextualized using continental-crust abundances normalized to Au on a base-10 logarithmic scale (as indicated), with warmer shading denoting higher relative abundance.

In view of these advances, we argue that MXenes are not only strong candidates to outperform many established materials in environmental applications, but also open new opportunities and enable previously unattainable technologies. At the same time, a rigorous and realistic evaluation of their potential, limitations, and sustainability implications is required. In this Perspective, we present an interdisciplinary, forward-looking view that links the unique properties of MXenes to specific sustainability challenges and translates these insights into a call to action for the community. Short- and long-term plans to tackle current research bottlenecks are identified, and applications where MXene-based materials and devices might offer viable solutions are outlined, hence consolidating their role as indispensable materials for a sustainable future.

## Precursor Availability and Sourcing

The path to the commercial application of MXenes rests on two interdependent factors: (i) securing an abundant and reliable supply of precursor materials, and (ii) achieving scalable, controllable, and high-quality synthesis.^38,49^ While the latter will be addressed in the following section, we begin by focusing on the availability of MXene precursors, as summarized in Figure 1.

Using abundant, geographically distributed, and inexpensive elemental precursors reduces vulnerability to supply disruptions, besides opening the way for simpler and more energy/cost-efficient synthesis routes. Aluminum (Al) and titanium (Ti), primary elements for MAX/MXene systems, are common and inexpensive metals: Al is the second most abundant metallic element and constitutes ∼8.2 wt % Earth’s crust,^50^ with a price of ∼3.92 USD kg^–1^ in June 2025;^51^ Ti is likewise accessible, with an abundance of ∼0.38 wt %,^51^ but slightly more expensive (average of ∼10 USD kg^–1^ in the second quarter of 2025 ^52^). Al scrap, TiO_2_, and Ti sponge can be used to further reduce the production cost and environmental impact of MAX phase precursor synthesis.^53–55^ This natural abundance supports large-scale primary production, not to mention the mature recycling infrastructure for Al, with developing branches for Ti. The case for scalability extends to other common MXene precursors, such as zirconium, vanadium, niobium, molybdenum, and silicon (carbon and nitrogen are excluded from this analysis due to their ubiquitous and diverse global supply). These elements are already established commodities in the global industry with well-developed extraction networks and, in many cases, efficient recycling protocols in place (see Box 1).

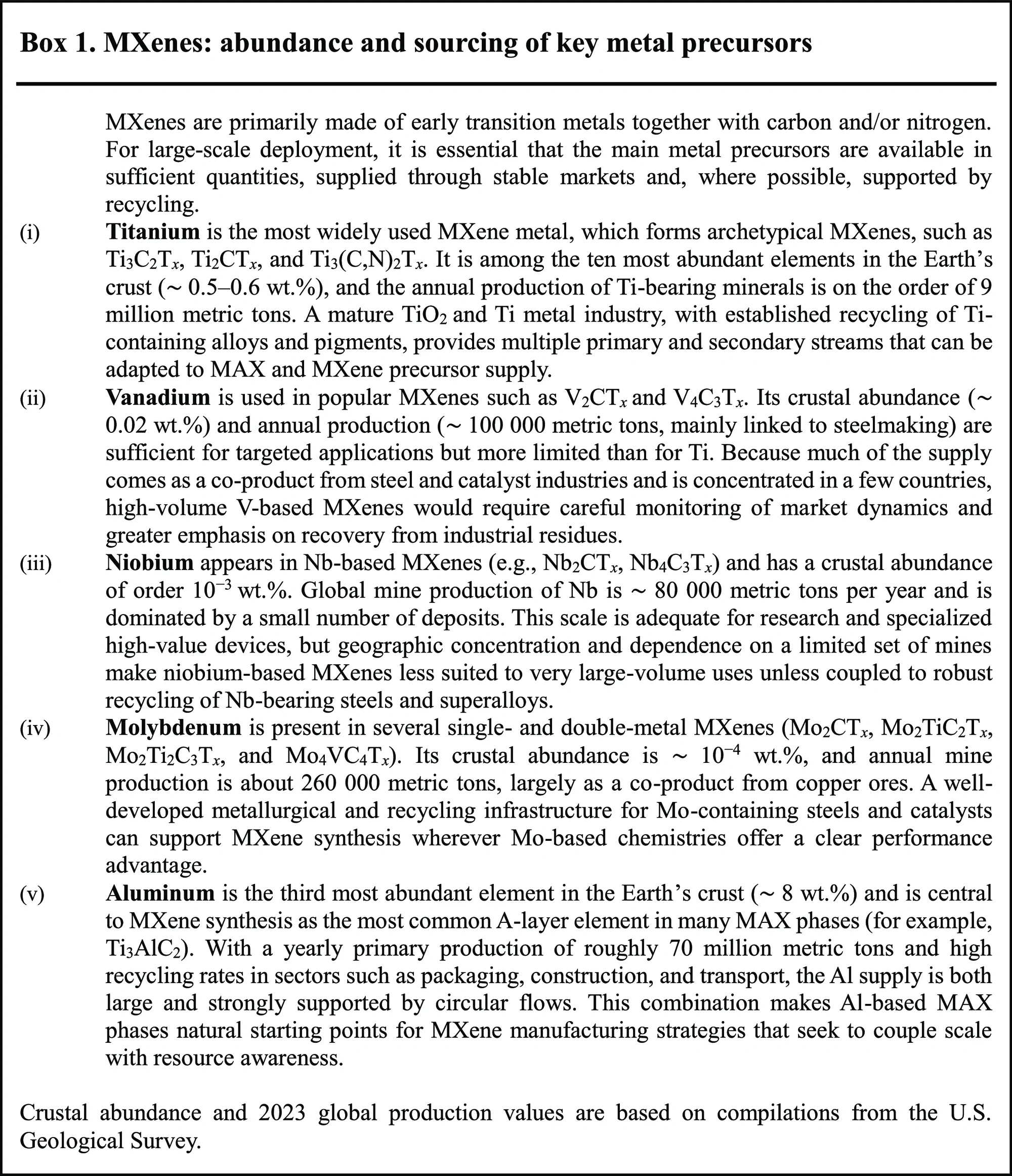



MXene precursors typically occur as minerals and are processed into metallic or oxide powders through established industrial routes. Either form can be used as a feedstock for MXene production, depending on the target chemistry and the chosen synthesis method. Oxides, in particular, allow for the use of low-cost ores and recycled materials, which have already proven effective in producing high-quality MXenes by self-propagating high-temperature synthesis or conventional ceramic technology, promoting the circular economy and reducing the environmental footprint of synthesis.^55,56^ These considerations make large-scale production of MXenes unlikely to be constrained by elemental scarcity alone, which is a first step toward sustainability credentials. However, the full assessment is complex and must also account for water consumption, energy use, waste generation, scalability, toxicity, and yield to determine whether a given MXene technology offers an environmental advantage over established materials and processes. Such supply chain considerations are crucial for future large-scale applications, especially in energy storage, water desalination, and thermal management, where there is demand for substantial volumes of advanced materials.

While the compositional design space of MXenes is broad and most constituent elements are abundant and industrially available, synthesis routes often pose significant environmental and safety concerns. The development of reliable and scalable synthesis methods is central to realizing the potential of MXenes. Life-cycle assessments of Ti_3_C_2_T_*x*_ produced via conventional HF-based selective etching, for instance, point to energy-intensive processing as well as environmental health and safety (EHS) risks associated with HF and related hazardous reagents.^57–59^ These concerns have accelerated efforts toward HF-free and more energy-efficient routes, including molten-salt, gas-phase, and direct synthesis, besides environmentally friendly delamination protocols.^38,60^ The sustainable synthesis of MXenes is therefore an active and critical frontier in the field, with an emphasis on greener, less hazardous, and more cost-effective methods, which consume less resources, such as water.^61^ The central question now is scale: can these approaches be industrialized without simply trading HF for other energy, chemical, or waste penalties? That question motivates the manufacturing perspective that follows.

## From Lab Synthesis to Green and Scalable Manufacturing

As MXenes transition from laboratory-scale preparation to scalable manufacturing, synthesis must be evaluated not only in terms of phase purity and yield, but also through a broader framework that accounts for environmental impact, operator safety, and resource intensity. Figure 2a summarizes the current synthesis landscape, spanning direct CVD growth and production from MAX phases and related precursors via diverse etching routes (wet-chemical, electrochemical, molten-salt, hydrothermal, and gas-phase) and downstream delamination steps that ultimately determine flake quality and process throughput. Crucially, Figure 2b illustrates how these routes have emerged and diversified over time and provides a qualitative, expert-informed radar-chart comparison across key criteria (water and energy use, yield, waste generation, toxicity, and scalability), based on input from both leading academic researchers and commercial MXene producers, thus offering a broad overview of the current state-of-the-art methods. Delamination was not evaluated as a separate category in these comparisons, except where it is intrinsically embedded in a given synthesis route. Together, these panels frame the central challenge for the field: preserving MXene performance while shifting synthesis toward safer, scalable, environmentally responsible, and resource-efficient manufacturing.

**Figure 2:**
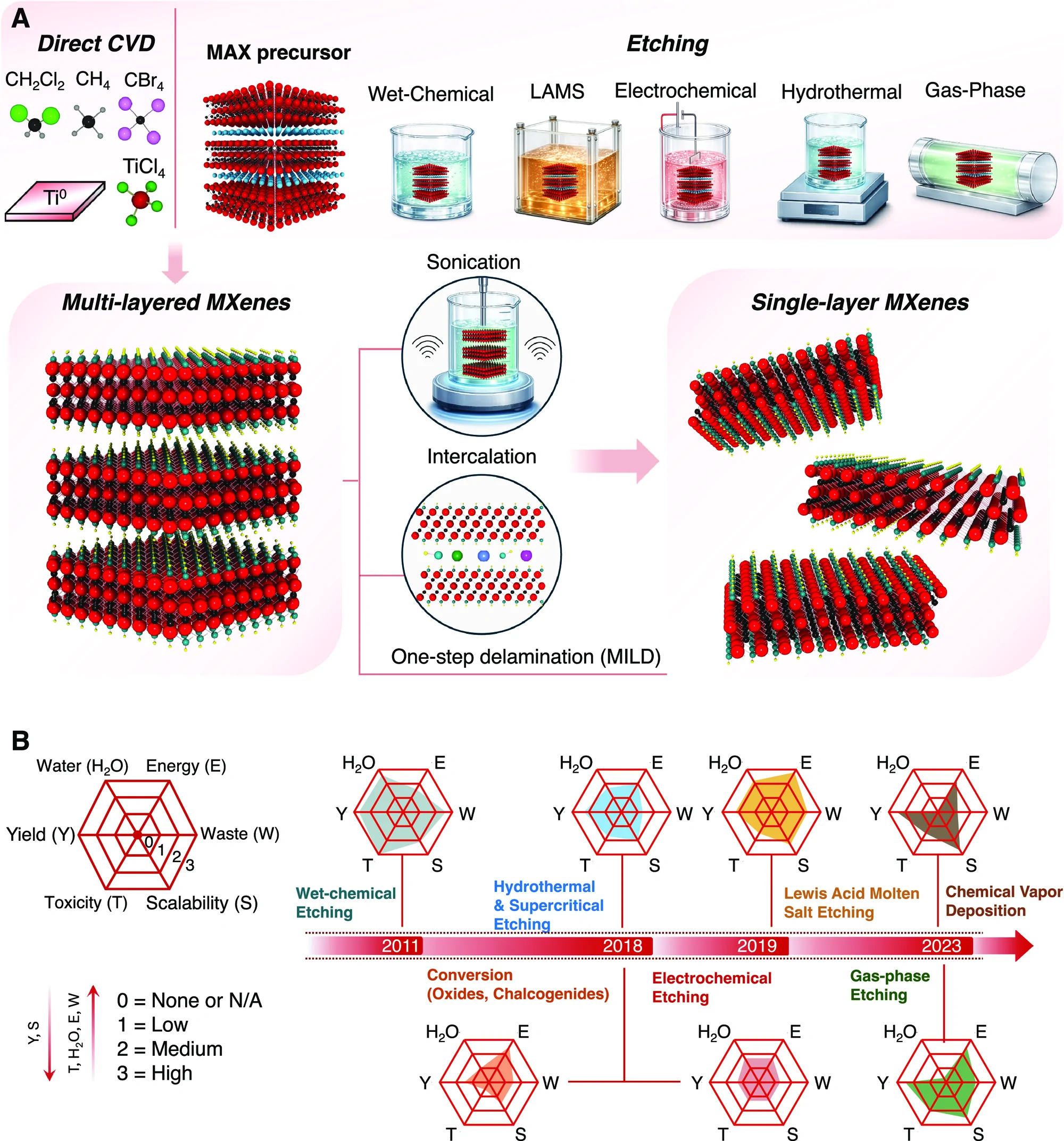
Synthesis routes to multilayer MXenes and their conversion to single-layer flakes and sustainability trade-offs. (A) Schematic overview of two entry points to multilayer MXenes: direct synthesis (e.g., chemical vapor deposition (CVD) growth from simple precursors) and selective etching-based synthesis starting from layered MAX precursors, illustrated for wet-chemical, Lewis acidic molten-salt (LAMS), electrochemical, hydrothermal and gas-phase etching routes. These routes converge on multilayer MXene stacks (left), which are subsequently converted into single-layer MXene flakes (right) through delamination enabled by intercalation, sonication, or via one-step synthesis approaches such as minimally intensive layer delamination (MILD). (B) A comprehensive timeline and qualitative radar-chart comparison of major MXene synthesis routes. Each radar chart scores a given route on six axes – water consumption (H_2_O), energy usage (E), waste (W), scalability (S), toxicity (T), and yield (Y) – using a discrete 0–3 scale (0 = none or not applicable; 1–3 = increasing level, with the W axis reflecting both amount and hazard of waste streams). Scores are based on expert assessment and are intended to highlight trade-offs and sustainability trends across synthesis methods rather than provide a strict ranking.

The vast majority of MXenes are produced via top-down selective etching of MAX phases and related compounds (Figure 2a) using concentrated hydrofluoric acid.^17,38^ In the qualitative expert assessment (Figure 2b), wet-chemical HF etching is consistently rated as very high in toxicity and generally high in waste generation, alongside substantial water consumption driven by repeated post-etch washing and separation steps. Although effective and applicable to a variety of carbide and carbonitride MXenes, hydrofluoric acid is corrosive and toxic, requiring stringent safety protocols and generating fluoride-containing waste streams that need neutralization and disposal.^59^ Furthermore, the etching process results in mixed and poorly controlled surface terminations, especially fluorine-based species, which may complicate environmental risk assessments, as hydrolysis of Ti–F bonds produces HF when MXenes are stored in water or used in aqueous media. At the same time, wet-chemical etching is perceived as comparatively favorable for manufacturability, with high yields and good scalability, reflecting the fact that the main burden comes from chemical hazard management and downstream water-intensive processing. This imbalance (strong manufacturability but an unfavorable toxicity/waste profile) has motivated modifications of the standard wet-etch route that preserve yield while reducing handling risks and, ideally, fluorine incorporation.

The Minimally Intensive Layer Delamination (MILD) method replaces HF with a fluoride salt (typically, LiF or NaF) in combination with HCl, improving operational safety and reducing the fraction of F-terminations.^31^ However, from the radar-plot perspective, it still falls within a wet-chemical processing envelope, where toxicity and waste are dominated by fluoride handling and disposal, and where water demand has yet to be reduced. This milder etchant leads to an easy-to-delaminate product but cannot dissolve grain boundaries in MAX phases and is not aggressive enough to etch some of the most stable MAX precursors. Related approaches that add a small HF fraction to HCl can minimize the amount of F-terminations and reduce etching time, being applicable to a larger number of MAX phases.^38^ NH_4_HF_2_ solution provides the fastest etching at room temperature,^62^ and supercritical CO_2_ can further speed up the process,^39^ yet these variants still generate fluoride-containing waste streams. Electrochemical etching and etching in supercritical water, mechanochemical routes, microwave heating and solvent-minimized strategies represent an additional frontier in sustainable MXene synthesis, but those need to be optimized to achieve high quality and yield. Repeated washing and centrifugation steps, required after selective etching in F-containing solutions and delamination, consume large volumes of water and energy, increasing the overall environmental footprint.^49^ For large-volume production, these steps should be replaced by filtration, dialysis, and spray-drying to reduce liquid waste and simplify recovery, an especially impactful target given the elevated H_2_O scores assigned to wet-chemical processing.

Sustainability considerations extend beyond the etching step to encompass delamination, surface termination control and postprocessing. The chemical terminations acquired during synthesis strongly influence MXene properties, including electronic structure, hydrophilicity, and chemical reactivity. Conventional HF-based methods produce mixed F, OH, and O terminations. From both performance and environmental perspectives, reducing fluorine content is desirable. Postsynthesis treatments such as annealing in controlled atmospheres, electrochemical oxidation or washing in an alkaline solution have been used to tailor surface chemistry toward oxygen- and hydroxyl-rich terminations. Delamination producing single- or few-layer MXenes requires intercalation of cations and subsequent washing. While Li or Na ions can be used to delaminate Ti_3_C_2_T_*x*_, less environmentally friendly tertiary amines are used to delaminate other than Ti-based MXenes.^17^ These additional steps introduce extra energy/water consumption or chemical inputs, complicating lifecycle assessments. Developing one-step processes that inherently produce easy-to-delaminate MXenes (similar to MILD synthesis) with environmentally benign terminations remains an important research objective.

In response to these concerns, substantial experimental efforts have been directed toward developing fluoride-free and less hazardous etching strategies. One promising route involves selective etching of the A element in Lewis acidic salts such as ZnCl_2_ or CuCl_2_.^40,41^ In the expert scoring (Figure 2b), LAMS shifts the perspective: toxicity is rated low-to-moderate, and water use is typically medium because salt removal and washing are still required. However, energy demand is rated high due to sustained elevated temperatures, while waste is assessed as intermediate and more variable (“W” spanning 1–3 depending on salt recovery and byproduct handling). Importantly, LAMS is viewed as maintaining good manufacturability, consistent with its process robustness and the broad industrial familiarity with molten-salt operations. Related halogen-based gas-phase etching strategies, using halogen-containing compounds (TiCl_4_, HCl, etc.^42,43^ ) also avoid HF but with a distinct trade-off profile: experts emphasize very low water demand in the core process (H_2_O can approach 0) together with high yield and scalability, while toxicity and waste are generally rated moderate and depend on halogen handling and capture. Besides eliminating the need for hydrofluoric acid, both LAMS and gas-phase etching enable more controlled and uniform halogen surface chemistries (single-species halogen surface terminations). Those methods can be energy intensive, but molten salts are widely used in industry and halogen etching of carbides is also used at the industrial scale for producing carbide-derived carbons, as well as silicon and boron chlorides.^63^ A remaining challenge in the LAMS process is postsynthesis purification, particularly at scale, since residual salt removal must be achieved without increasing environmental impacts related to water use or waste handling.

CVD synthesis of MXenes (Figure 2a) provides an alternative that bypasses MAX-phase etching and instead directly grows ultrathin transition metal carbides or nitrides on substrates or in the vapor phase from metal halogenides and gaseous carbon and nitrogen sources.^44–46^ In this method, volatile metal halogenide precursors react with carbon- or nitrogen-containing gases (e.g., CH_4_, NH_3_) at elevated temperatures to form halogen-terminated M–X layers. Consistent with the expert scoring (Figure 2b), CVD is associated with low water use and relatively low solid/liquid waste, along with good yield and high scalability potential. Nonetheless, it involves a relatively high energy demand due to the thermal requirements for vapor-phase growth. Similar to molten salt and halogen etching methods, CVD enables precise control over surface terminations, but the process is currently limited to M_2_C and M_2_N structures. Since the synthesis of T_2_CCl_2_ is possible using TiCl_4_ (an intermediate product for TiO_2_ production) or related reduced derivatives and methane (natural gas) in a fluidized bed process,^44^ its production volume and cost may eventually reach that of TiO_2_ powder. While still less developed than etching-based methods, gas-phase growth offers a promising pathway toward low-cost synthesis of high-purity and highly stoichiometric MXenes.

It is important to mention that all the above methods diverge into the synthesis of hydrophilic O/OH-terminated and hydrophobic halogen-terminated MXenes. The former are easy to delaminate and disperse in water or polar organic solvents. Delamination of the latter, however, is challenging,^60^ and they may therefore be suited for applications where powders rather than single flakes are required, such as battery electrodes or sorbents. Halogen terminations also allow easier chemical modification and covalent bonding of organic molecules.^18^

Theoretical studies have played a pivotal role in guiding sustainable MXene synthesis. Density functional theory (DFT) calculations have been widely used to evaluate the energetics of A-layer extraction,^64^ predict stable surface terminations,^19^ and assess the influence of the chemical environment on reaction pathways.^64–66^ By calculating reaction barriers and binding energies, computational models help identify etchants that minimize energy requirements while preserving structural integrity. Simulations have also elucidated how different terminations alter electronic properties,^67^ enabling rational design of synthesis conditions that yield target functionalities without extensive empirical trial and error. Molecular dynamics simulations further contribute by modeling intercalation, delamination, and defect evolution processes,^68^ particularly under nonequilibrium conditions such as mechanical milling or electrochemical bias. Such theoretical insights reduce experimental redundancy and resource consumption, thereby indirectly supporting sustainability goals. While fundamental studies and high-throughput screening have to date primarily relied on DFT, machine learning (ML) approaches are increasingly being applied to predict both stability and functional properties.^69^ Examples aligned with sustainability objectives include MXenes for energy storage applications,^70^ CO_2_ activation,^71^ and MXene-based single-atom catalysts,^72^ suggesting that AI-assisted materials development will play a crucial role in the design of future MXenes with targeted properties and performance, as well as in accelerated identification of synthesis pathways with low environmental impact.

Despite impressive progress, remaining obstacles hinder the realization of fully sustainable MXene production. Achieving high yield and phase purity with fluoride-free aqueous etching methods remains challenging, especially for less common MAX precursors. Controlling defect density while maintaining scalable throughput is another persistent issue. Wastewater management, including recovery of dissolved metal ions and recycling of process water, is often overlooked but critical for industrial viability. Moreover, the energy demands of drying, delamination, and post-treatment steps can offset gains made by greener etching chemistries. Lifecycle assessments comparing different synthesis routes are lacking, and standardized metrics for environmental impact are needed to meaningfully evaluate progress.

Looking forward, the convergence of experimental innovation, theoretical modeling, and systems-level sustainability analysis will be essential. Recyclable etchants and closed-loop processing systems could mitigate waste generation. Data-driven methods, including machine learning models trained on computational and experimental data sets, may accelerate the discovery of greener reaction conditions. Ultimately, the sustainable synthesis of MXenes is not merely a matter of replacing one etchant with another, but of rethinking the entire production chain from precursor fabrication to end-of-life considerations. Addressing these challenges will determine how quickly MXenes can transition from the current small-batch synthesis^46^ to mass production and become environmentally responsible components of next-generation technologies.

Beyond the chemistry of etching and delamination, storage, processing, and end-of-life behavior also require attention. Processing from aqueous dispersions with no surfactants makes MXene inks attractive compared with many carbon- and metal-based conductive inks, but MXenes can be vulnerable to oxidation and hydrolysis during storage. Degradation rates depend on the quality of the material and on washing, drying, storage conditions, concentration, and formulation, with direct implications for shelf life. For Ti-based MXenes, oxidation and hydrolysis can yield TiO_2_-containing degradation products, with chemistries that vary according to residual surface terminations, synthesis byproducts, additives, and the degradation environment. MXene–polymer composites, fluorinated surfaces, and halogen-rich materials introduce additional separation, recovery, and disposal challenges and therefore require formulation-specific recycling or end-of-life protocols.

Several strategies can address these challenges, including proper handling, quality control, and storage-specific mitigation approaches such as refrigeration of concentrated MXene dispersions.^73,74^ Although further work is needed to improve stability without introducing new environmental concerns, common MXenes, such as T_3_C_2_T_*x*_ or Nb_4_C_3_T_*x*_, can be more stable than many other nanomaterials under appropriate conditions. Their stability can also be tuned by composition, surface chemistry, formulation, and storage environment, which may be useful either for extending service life or, in selected cases, for promoting faster degradation after disposal. If stability is managed by design and, where needed, oxidized material can be recovered or reconditioned, MXenes can deliver outstanding performance in many sustainability-relevant technologies.^75^

A careful assessment of precursors, synthesis routes, and stability is fundamental to understanding the practical limits of MXene technologies. Only materials that can be produced at scale with controlled quality and an acceptable environmental footprint will move beyond laboratory synthesis. For commercialization, however, the relevant metric is not simply the amount of MXene that can be produced, but whether a given route delivers reproducible material at a competitive cost per function. This depends on precursor grade and price, conversion yield, material reproducibility, postprocessing cost, shelf life, and compatibility with industrial setups and composite-manufacturing workflows. Chemically and environmentally elegant routes that produce low yields, broad property variation, difficult-to-remove residues, or unstable dispersions will face major barriers to adoption. In contrast, more energy-intensive routes may become attractive if they provide high yield, high purity, controlled terminations, reduced wastewater burden, and direct compatibility with existing infrastructure. Here, through the expert-informed radar plot (Figure 2b), we compare leading routes and their trade-offs, highlighting where cleaner, more efficient production is being developed and where bottlenecks remain: wet-chemical processing remains strong in yield and scalability but is penalized by toxicity, water use, and waste; LAMS, gas-phase etching, and CVD reduce chemical hazards and wastewater but introduce higher thermal-energy demands; and emerging electrochemical, hydrothermal, and conversion strategies score favorably on several axes but still require breakthroughs in throughput and robustness (Figure 2b). Based on that, we now turn to key applications where MXenes already offer clear sustainability advantages in real devices and systems.

## Key Applications that Can Contribute to Our Sustainable Future

As synthesis routes mature and production capacity grows, MXenes are moving from lab-scale materials to active components in devices with direct relevance for energy use, environmental protection and human health (Figure 3). Their value in these areas goes beyond simply replacing established materials and includes addressing problems that conventional materials solve only partially. Competing materials might remain preferable in many contexts because they are cheaper, more mature, environmentally stable, or already embedded in industrial supply chains. Nonetheless, MXenes become compelling when multifunctionality and tunable properties are needed in a single component, offering not only enhanced performance but also the potential to reduce material use, processing complexity, device weight, or component count while maintaining sufficient durability and recyclability. With this perspective, we next identify a set of key applications in which MXenes offer tangible advantages over conventional materials and discuss the main bottlenecks to practical deployment.

**Figure 3:**
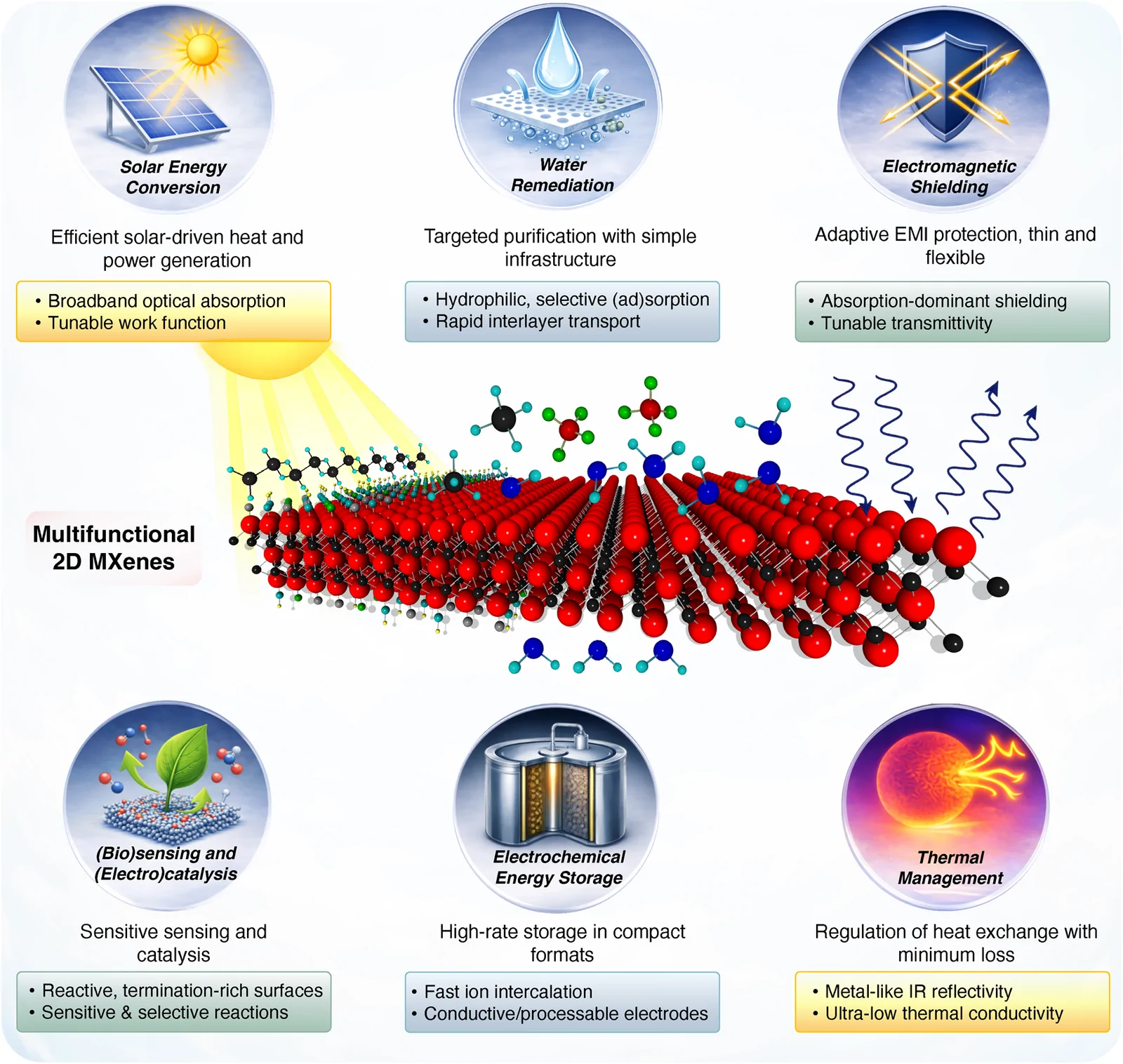
MXenes as a multifunctional platform for sustainability-relevant technologies. A partially terminated, solution-processable M_3_X_2_ MXene sheet is shown at the center, illustrating how metallic conductivity, tunable surface terminations, and rapid interlayer transport can be leveraged across diverse device concepts. Surrounding icons highlight the key application areas discussed in this Perspective: thermal radiation management, solar energy conversion and solar-driven water remediation, electrochemical energy storage, (bio)­sensing and (electro)­catalysis, and electromagnetic/radiation shielding. Across these domains, the same core MXene attributes can translate into lower energy demands, scalable coatings and devices, and reduced reliance on critical raw materials.

### Thermal Management

Effective thermal management is central to several human activities, from the clothing we wear to the industrial equipment we use and buildings we inhabit, being thus a core component of technologies across diverse fields. Nonetheless, despite the constant development of multiple active and passive thermal management technologies, controlling the associated energy consumption/loss remains challenging. In contrast to external energy modulation (e.g., air conditioning), radiative thermal management can regulate heat exchange by tuning infrared (IR) emissivity and absorptivity, which improves the energy exchange efficiency between objects with different temperatures with no additional energy cost and with low heat waste. Metallic MXenes combine metal-like IR reflectivity with ultralow thermal conductivity. Ti_3_C_2_T_*x*_ films, for example, exhibit an out-of-plane thermal conductivity of about 0.2 W m^–1^ K^–1^,^76^ orders of magnitude lower than metals and much lower than typical 2D materials like graphene and transition metal dichalcogenides. At the same time, Ti_3_C_2_T_*x*_ reflects mid-IR radiation like a polished metal, which leads to roughly 2 orders of magnitude lower radiative heat loss than common metal foils.^77^ It is important to note that these thermal and optical properties depend strongly on the synthesis route, flake size and quality, and surface terminations. Moreover, oxidation or hydrolysis can alter conductivity, infrared emissivity, and photothermal response, making stability a critical point. However, this combination of properties makes it possible for submicrometer MXene coatings to act as thermally insulating heat barriers, opening new opportunities for ultrathin insulation and efficient thermal management. MXene can be spray-coated from aqueous dispersions with no additives onto any surface, including building walls, which makes their application easy and facilitates their widespread use.

In personal thermal management, MXene-decorated smart textiles have been realized by spraying Ti_3_C_2_T_*x*_ nanosheets onto polyester/polyurethane fabrics, which raises the average temperature of the fabric by ∼7 °C compared with conventional materials such as cotton, and increases skin temperature by ∼2–3 °C using only body heat as the energy source.^78^ Research has shown that such personal thermal management strategies may significantly contribute to reducing energy consumption when compared to conventional wearable heating and cooling garments.^79,80^ At the building scale, operational energy use accounts for about 30–40% of global final energy consumption, a significant portion of which is attributed to thermal losses and gains through walls and windows.^81^ The development of “smart windows” that dynamically modulate solar and IR transmission (such as electrochromic and polymer-dispersed liquid-crystal (PDLC) films) can reduce annual heating, cooling, and lighting demands by roughly 20–30% compared with static glazing, depending on climate and control strategy.^82^ Transparent Ti_3_C_2_T_*x*_-based conducting electrodes have already been tested in flexible PDLC smart windows and offered high optical transmittance, good conductivity, low operating voltages, and robust bending stability.^83^ When combined with smart window architectures, MXenes offer a realistic direction toward durable and potentially lower-cost dynamic glazing that can reduce energy consumption.^81,84^ For practical integration, however, into textiles, windows, and other exposed surfaces, more systematic and standardized evaluation is required for coating adhesion, abrasion resistance, washability, humidity tolerance, and stability under repeated thermal cycling.

Taken together, these developments indicate that MXenes can support more energy-efficient and environmentally conscious solutions in applications where thermal radiation must be controlled. Their IR selectivity and strong light–to–heat conversion make them natural candidates for applications where radiative heat flows are controlled and not compensated by active heating or cooling. Similar photoinduced and selective behavior is fundamental in solar-driven energy conversion and water remediation, where harvesting is at the core of a successful device.

### Solar-Driven Energy Conversion and Water Remediation

Solar energy is a vital component of global decarbonization efforts and has driven decades of innovation in light-harvesting technologies. MXenes have recently attracted increasing attention as candidates for both solar-thermal and photovoltaic (PV) applications. This interest stems from their broadband optical absorption, efficient photothermal conversion, high electrical conductivity and tunable work function, which together make them suitable as active components in next-generation solar-energy devices.

Solar-driven interfacial evaporation is a representative case where MXene-based light-to-heat conversion directly supports sustainable water desalination. Randomly oriented Nb_2_CT_*x*_, Nb_4_C_3_T_*x*_, and Ti_3_C_2_T_*x*_ films and aerogels display exceptional solar absorption and highly efficient photothermal conversion. As a consequence, a thin MXene layer at the water/air interface can localize heat, raise the local temperature, and enhance evaporation while limiting heat losses to the bulk liquid.^85,86^ MXene-based evaporators combined with cellulose, polymer foams, or wood scaffolds can reach evaporation rates of ∼1.5–2.3 kg m^–2^ h^–1^ under one-sun illumination, with reported solar–thermal conversion efficiencies in the 85–96% range.^87–90^ Such designs have been efficient not only for desalination of seawater and hypersaline brine, but also for removal of organic contaminants and bacteria when MXenes are integrated with antibacterial components such as Ag nanoparticles or chitosan. In practical terms, these systems offer compact and low-infrastructure routes to clean water for off-grid or coastal communities using MXene devices made of Earth-abundant elements instead of noble metals. However, long-term operation in real water streams is considerably less explored than performance under controlled laboratory conditions. Variables like salt accumulation, organic and biological fouling, and oxidative degradation can compromise water flux, ion selectivity, and photothermal performance. In addition, interlayer swelling, structural stability, and durability during repeated regeneration persist as important challenges for long-term deployment and operation.

Beyond water remediation, MXenes are being explored in integrated strategies that couple solar steam generation with “blue-energy” harvesting from salinity gradients.^91^ A recent example is a photothermal gel-cotton evaporator in which Ti_3_C_2_T_*x*_ and liquid-metal particles convert sunlight into heat for seawater evaporation. Besides efficient desalination, the resulting salinity gradient could still be used to power an osmotic generator with power densities on the order of 1 W m^–2^ under one-sun operation.^92^ In parallel, Ti_3_C_2_T_*x*_ and oxygen-functionalized MXene membranes with well-defined nanofluidic channels have displayed high ion selectivity and osmotic power densities from a few to several tens of W m^–2^ under seawater-river water or NaCl gradients, confirming their suitability as active elements in salinity-gradient energy harvesters.^93–95^ Solar–osmotic architectures thus offer a plausible solution to produce freshwater and electricity simultaneously from the same footprint, improving the use of coastlines and industrial brine without additional fossil energy input.

In photovoltaics, MXenes (most prominently Ti_3_C_2_T_*x*_) are now integrated at several positions within the PV device, including transparent electrodes, carrier-transport layers, and interfacial additives. This progress has been mapped across perovskite, organic, and silicon solar cell technologies, considering MXenes’ unique combination of high conductivity and adjustable work function as main advantages. As transparent electron transport layers, Ti_3_C_2_T_*x*_ films and hybrids can replace indium tin oxide and noble metals in silicon heterojunctions and perovskite solar cells, maintaining high optical transmittance and supporting power-conversion efficiencies above 22–24% for large-area Si cells.^96^ In perovskite and organic solar cells, in turn, Ti_3_C_2_T_*x*_ might be employed as an additive in the absorber, as well as in electron- and hole-transport layers where it improves film crystallinity, boosts band-edge alignment and suppresses nonradiative recombination. Device efficiencies approaching or exceeding 20% with enhanced operational stability have been reported for different systems.^4,97,98^

MXene-based solar absorbers, evaporators and solar cells illustrate how MXenes can link sunlight harvesting with both electricity generation and clean-water production. Strong solar-to-heat conversion allows for compact interfacial evaporators that desalinate or remediate water under natural sunlight, with limited thermal losses to the bulk. In PV and solar–osmotic architectures, efficient electronic and ionic transport through MXene layers helps to reduce resistive and interfacial losses, and MXene electrodes can mitigate the dependence on indium-based transparent conductors and noble metals. Interestingly, the same capacity to move electrons and ions through stacked 2D nanosheets and across redox-active surfaces lies at the core of MXene-based electrochemical energy storage described below.

### Electrochemical Energy Storage

A defining contribution of MXenes is the introduction of high-rate pseudocapacitive energy storage. Unlike traditional carbon supercapacitor electrodes that rely mainly on electrostatic charge storage, MXenes can store charge through fast surface redox reactions on their large and open surface, while maintaining metallic conductivity during the redox cycle. This may enable devices that combine battery-like energy density with capacitor-like power density. For example, Ti_3_C_2_T_*x*_ negative electrodes have demonstrated volumetric capacitances approaching ∼1500 F cm^–3^,^5^ capacitance retention of 92% after 500,000 cycles,^99^ and operation at scan rates exceeding conventional carbon supercapacitors in protic electrolyte.^31,32^ Translating this performance to thick, practical electrodes is still challenging because restacking can restrict ion accessibility at high mass loadings, and oxidation might progressively reduce electrical conductivity and electrochemical activity. Nevertheless, such performance established MXenes as a benchmark for high-power energy storage and enabled the development of microsupercapacitors, flexible devices, and fast-charging systems that may be of particular importance for data centers powering AI and for grid stabilization.

In battery research, MXenes have been tested as high-capacity anodes and conductive hosts for Li-ion and emerging ion chemistries.^100^ Theoretical capacities of 390–600 mAh g^–1^ and experimental reversible capacities exceeding 400 mAh g^–1^ have been demonstrated, but this may not be sufficient to compete with commercial silicon–carbon anodes. MXene’s tunable interlayer spacing enables efficient ion intercalation for Na^+^, K^+^, Zn^2+^, and other ions, making them candidates for anodes of beyond-Li batteries.^3^ Suppression of dendrites allows the use of MXenes as current collectors of anode-free metal batteries. Replacing thick and heavy copper current collectors with 3–4 times thinner and an order of magnitude lighter free-standing micrometer-thick MXene films with a strength of aluminum may well become the first and most promising battery application of MXenes.^101^ The use of Ti-based MXenes as electronically conductive additive/binder to replace carbon black and PVDF in Si and graphite anodes or metal oxide cathodes may increase the power of a variety of batteries. Still, full-cell performance, industrially relevant mass loadings, scalable manufacturability, and end-of-life recovery have yet to be sufficiently demonstrated.

Beyond performance metrics, the tunability and variety of MXenes, solution processing into films and inks, and integration into flexible and biodegradable devices contribute new design paradigms in energy storage. From a sustainability perspective, electrochemical energy storage based on MXenes offers several advantages: fast charge–discharge at modest voltages, compatibility with aqueous or low-toxicity electrolytes, the use of relatively abundant elements in many leading compositions, and the possibility of processing into thin films, coatings and printable inks for compact or flexible devices. At the same time, challenges remain in stabilizing MXenes against oxidation/hydrolysis and designing electrodes that can be recycled and not landfilled. Progress will depend on coupling device optimization with life-cycle assessment and on developing recovery strategies that avoid shifting the environmental burden elsewhere in the energy chain. Beyond storage, the coupled electron/ion-transport mechanisms and surface chemistry can be explored for chemical transduction, forming the basis for MXene-based sensing and catalytic activity.

### (Bio)­Sensing and (Electro)­Catalysis

Sensing and catalysis affect nearly every aspect of a sustainable society. Sensors that detect biomarkers, contaminants or environmental analytes support preventive healthcare, food safety and pollution control. Electrocatalysts in turn mediate the electrochemical conversion of water, nitrogen and carbon dioxide into fuels and chemicals. MXenes are attractive for these applications because their metallic conductivity facilitates fast electron transport, and their hydrophilic, chemically tunable surfaces ensure a high density of distinct active sites for adsorbate binding and catalysis. Unlike many chemically inert 2D materials that require defect engineering before they become active, MXenes offer intrinsically active interfaces that couple reliable electrical transport with selective chemical interaction. These attributes have driven intense research into MXene-based bio and chemical sensors and (electro)­catalysts as candidates for higher performance at lower material and environmental cost.

Biosensors that incorporate MXenes have rapidly evolved and are redefining performance benchmarks for environmental monitoring and noninvasive healthcare diagnostics. In addition to their high conductivity, the major advantage of MXenes over commonly used materials such as graphene lies in their conductivity and solution processability (particularly in water) and diverse surface chemistry, which together promote higher sensitivity and lower operating potentials in electrochemical biosensors.^102,103^ In the context of environmental sustainability, for example, TiO_2_-decorated Ti_3_C_2_ MXene achieved hydrogen peroxide detection down to 14 nM, a detection limit compatible with trace-level monitoring for environmental safety.^104^ Gas sensing investigations have shown that surface-engineered Ti_3_C_2_T_*x*_ and V_2_CT_*x*_ can detect toxic gases (e.g., NH_3_, NO_2_) and volatile organic compounds at room temperature with limits of detection in the ppb range.^105^ In water monitoring, MXenes serve as matrices for biocompatible high-performance electrochemical and optical sensors targeting pesticides, phenols and heavy metal ions (e.g., Pb^2+^, Cd^2+^) in complex aqueous environments, often outperforming standard carbon or metal-oxide platforms.^106,107^ However, application to real biological and environmental samples continues to be constrained by long-term signal stability, insufficient selectivity, and interference from complex sample matrices. These challenges may be further affected by defect chemistry and oxidation state during operation, which can influence, for example, calibration and measurement reproducibility.

MXene-based inks and coatings on textiles or elastomers is also a rapidly growing research area given the possibility to achieve low-voltage, on-body sensing of sweat metabolites, pressure and strain, supporting healthcare models built on decentralized and continuous monitoring.^102,108^ Fabricated via scalable water-based methods, MXene electrode arrays (“MXtrodes”) have performed better than commercial gold and Ag/AgCl electrodes in electroencephalography (EEG), electromyography (EMG), and electrocorticography (ECoG) applications.^109^ We believe that the immediate focus must prioritize the engineering of robust and reusable electrodes for electrophysiology and sensors from these prototypes, ensuring functionality against the exposure to complex real-world environment.

Parallel to sensing, MXenes have also been widely explored in the (electro)­catalysis field, where their physicochemical properties allow them to act both as intrinsically active phases (e.g., Mo_2_CT_*x*_ and V_2_CT_*x*_) and as key components in catalytic devices. By controlling MXene composition (single- versus multimetal), MXene order (*n*) and layer thickness, surface terminations, and defect chemistry, researchers have approached near-optimal adsorption energetics for environmental remediation and clean-energy catalysis.^110–112^ One example is the introduction of metal vacancies in Mo_2_TiC_*x*_ or Ti_3_C_2_T_*x*_ followed by anchoring single platinum atoms at the vacant sites, which produces single-atom catalysts (SACs) with exceptional hydrogen evolution reaction (HER) activity.^113^ More generally, MXenes provide a highly conductive 2D support that can immobilize SACs and clusters of adatoms or transition-metal complexes, acting synergistically to improve catalytic efficiency. Beyond HER, MXenes have shown activity in oxygen evolution and reduction reactions (OER/ORR), as well as in the electroreduction of CO_2_ and N_2_, serving either as the catalytic phase (especially nitride MXenes) or as conductive, redox-active supports.^114^ For practical electrocatalysis, however, the identity and evolution of the active sites are still insufficiently resolved in many systems. Similarly, factors such as surface reconstruction, leaching or migration of supported active species, catalyst poisoning, and stability at practically relevant current densities also require more systematic evaluation.

In environmental catalysis and remediation, MXene-based devices are now tested for the degradation of persistent pollutants like per- and polyfluoroalkyl substances (PFAS) within advanced water-treatment schemes.^115,116^ Owing to abundant surface functional groups, contaminants can be not only captured but, when feasible, also chemically transformed, e.g., by reducing toxic metal ions to less harmful forms, or by degrading antibiotics.^117^ Furthermore, MXenes maintain strong sorption capacity for heavy-metal ions and dyes in water, with reports of *>* 95% removal of Pb, Hg and organic dyes from contaminated water using MXene-based filters or membranes.^118^

Priority directions now include conducting systematic durability tests under realistic operating conditions, performing comprehensive life-cycle and techno-economic analyses against currently used catalysts, and designing MXene-based catalyst devices that can be regenerated or recycled. Research on (bio)­sensing and (electro)­catalysis does reveal how MXenes can contribute to cleaner water and air, along with lower-impact routes to fuels and chemicals, reducing the use of critical raw materials. When these systems rely on MXenes produced through greener synthesis routes, they can contribute to circular technologies and remain effective over long operating periods without frequent replacement or excessive energy input. The multifunctional features that benefit MXene-based (bio)­sensing and (electro)­catalysis are equally relevant for other applications such as in electromagnetic and radiation shielding in electronics, infrastructure and personal protection.

### Electromagnetic Interference Shielding

The densification of telecommunication systems and personal electronics has turned electromagnetic interference (EMI) into an inescapable form of pollution that threatens device reliability and raises biological safety concerns. EMI and other forms of unwanted radiation now affect everything from medical equipment and transportation to wireless communication and renewable-energy infrastructure. Conventional shielding typically relies on thick metals or metalized polymers that are heavy, prone to corrosion and difficult to recycle, especially when laminated with multiple polymer layers. This leads to high embodied energy and complex waste streams that sit poorly with the demands of next-generation sustainable technologies. MXenes offer an alternative based on thin, conductive, water-processable films and coatings that can be coated on or combined with lightweight polymers, foams and textiles.

Early work on Ti_3_C_2_T_*x*_ films reported shielding effectiveness above 90 dB in the X-band at thicknesses below 50 μm and 50 dB at 2 μm, showing that effective protection can be achieved with coatings far thinner than conventional metal foils.^119^ Follow-up studies demonstrated 20 dB (99%) shielding with transparent 40 nm Ti_3_C_2_T_*x*_ coatings. Laminated films and aerogel-like MXene composites that dissipate incident radiation mainly through multiple internal reflections and absorption instead of simple reflection also helped to limit secondary electromagnetic pollution.^120^ Many of these structures reach high shielding effectiveness at relatively low MXene loadings, so less active material is required for a given level of protection. Polyimide/MXene composites maintained shielding performance after severe thermal shock and exposure to chemically aggressive or high-temperature environments, extending service life and lowering the rate at which components become e-waste.^121^ More recent hybrid MXene-based materials incorporating polymers, carbon-based fillers, or magnetic nanoparticles have exhibited effective shielding from radio frequencies up to the terahertz regime, while maintaining low thickness and mechanical robustness suitable for mobile electronics, electric vehicles, and structural hardware.^122^ Importantly, direct comparisons across studies are difficult because shielding performance is often evaluated using different thicknesses, densities, frequency ranges, and relative contributions from reflection and absorption, which calls for standardized benchmarking protocols.

Sustainability considerations become even more evident in MXene-based textiles and flexible substrates. Micrometer-thin MXene coatings on cotton, silk or synthetic fabrics reach 30–50 dB EMI shielding in the GHz range (X-band) at low loadings and retain breathability and comfort for people who must wear protective garments for long periods.^120,123^ Additional functions such as Joule heating, strain sensing and even triboelectric energy harvesting have been demonstrated in MXene–textile systems. This could be relevant to the development of lightweight garments that provide EMI protection, thermal regulation and sensing simultaneously.^123^ At the device level, MXene–polymer films preserve shielding performance after repeated bending, stretching and exposure to harsh conditions.^121,124^ Such durability again helps to lower the overall materials and waste footprint by reducing the need for frequent replacement. Yet, humidity and oxidation or hydrolysis may reduce conductivity and shielding effectiveness, while film brittleness, coating adhesion, repeated-bending durability, and washing stability continue to be important design parameters.

MXenes can further be explored in multifunctional protection strategies that go beyond static EMI shielding. Electrochemically tunable MXene shields can switch between high attenuation and partial transmission through controlled ion intercalation and redox reaction, supplying strong protection only on demand and avoiding bulky permanent metal enclosures. In parallel, the intrinsically low infrared emissivity of titanium-based MXenes makes thin MXene coatings promising for thermal regulation of housings and electronics, reducing the need for active cooling. Coupled with flexible substrates and energy-harvesting modules in textiles, MXenes open a path to wearable covers that do more than shield, dynamically managing heat and providing power.^29^

Across coatings, foams and fabrics, MXene-based shields demonstrate that effective radiation protection can be achieved without heavy and rigid metal structures. Thin MXene layers can be processed from water-based inks and integrated into lightweight substrates, which lowers overall mass and reduces dependence on critical metals. Their high electrical conductivity and adjustable permittivity attenuate incident radiation mainly through absorption and multiple internal reflections, limiting secondary electromagnetic pollution. To move toward shielding technologies that are sustainable in practice, these advances need to be combined with HF-free or electrochemical synthesis routes, more oxidation-resistant MXene formulations and composite architectures designed so that components can be repaired, then separated and recycled at end of life.

## Material-Level Bottlenecks on Road to Deployment

Despite the promise outlined above, the deployment of MXenes will ultimately depend on how well their material-level limitations can be managed across synthesis, processing, operation, and end of life. A central challenge is that MXenes can be vulnerable to oxidation and hydrolysis depending on boundary conditions in aqueous dispersions, humid environments, and electrochemical or photothermal operation. Such degradation can lower conductivity, alter surface chemistry, change interlayer spacing, increase infrared emissivity, reduce sensing response, or modify catalytic activity. At the same time, the observed decade-long stability in the ambient laboratory environment clearly identifies the potential of MXenes for many applications. Stability should therefore be evaluated under application-specific environments rather than inferred from ideal storage or testing conditions alone.^73–75^

Surface-termination heterogeneity is another cross-cutting challenge. Wet-chemical etching commonly produces mixtures of O, OH, F, Cl, and adsorbed or intercalated species, with compositions that depend on precursor quality, etching route, washing, delamination, storage, and post-treatment. Even though molten-salt, gas-phase, and direct-synthesis routes can improve termination control, they may still produce heterogeneous surfaces through incomplete reactions, defects, residual species, or postsynthesis oxidation. Because terminations influence hydrophilicity, conductivity, work function, ion transport, and many other properties, small variations in surface chemistry can translate into large differences in device performance. More precise termination control and standardized reporting of composition, defects, flake size, oxidation state, and interlayer chemistry are therefore essential for reproducibility and industrial quality control.

These limitations become decisive under realistic operating conditions. MXene coatings, membranes, electrodes, sensors, catalysts, and shields must retain function under humidity, UV exposure, abrasion, fouling, oxidants, regeneration protocols, electrolyte exposure, bending, washing, thermal cycling, or long-term electrical bias, depending on the application. At the same time, MXenes must be benchmarked against established materials not only by peak performance, but also by cost-per-function, thickness- or mass-normalized performance, processability, durability, repairability, recyclability, and environmental footprint.^57,58^

The roadmap to transform MXenes from promising nanomaterials into deployable technologies is thus defined by these bottlenecks. Progress will require connecting synthesis chemistry to application-oriented degradation assessment, standardized benchmarking, techno-economic analysis, life-cycle assessment, and recovery or disposal protocols. MXenes offer distinctive advantages and are already used in selected applications, but broader deployment will depend on addressing the associated technical challenges in an integrated way.

One way to address several of these bottlenecks is to move beyond isolated MXenes and design them as stabilized, assembled, and hybrid architectures. Composite and hierarchical structures can improve mechanical integrity, environmental durability, processability, and multifunctionality, while also enabling integration with recyclable or bioderived components. This perspective naturally leads to viewing MXenes as modular building blocks for assembled nanomaterials, an approach that becomes especially compelling as far as sustainability is concerned.

## MXenes as Building Blocks for Assembled Nanomaterials

MXenes expand the library of two-dimensional building blocks that can be assembled into hierarchical structures. Because they are often processed from aqueous dispersions at room temperature or under other mild conditions, MXene flakes can be combined with polymers, biomacromolecules and additional nanomaterials to form layered films, fibers and three-dimensional lattices that are both mechanically robust and functionally active. This modular view is sketched in Figure 4, where MXenes appear alongside polymers, graphene, BN and transition-metal dichalcogenides as “nano-bricks” for sustainable technologies. Unlike purely passive reinforcements, MXenes are multifunctional materials that feature, among other properties, electrical/thermal conductivity together with redox activity and infrared response. Thus, assembled architectures have the potential to combine structural strength with energy storage, thermal regulation, or electromagnetic shielding within a single material platform. From a sustainability perspective, such assemblies offer a way to cut the number of separate components in a device, reduce processing energy and incorporate recyclable or bioderived constituents in materials designed for long service lifetimes.

**Figure 4:**
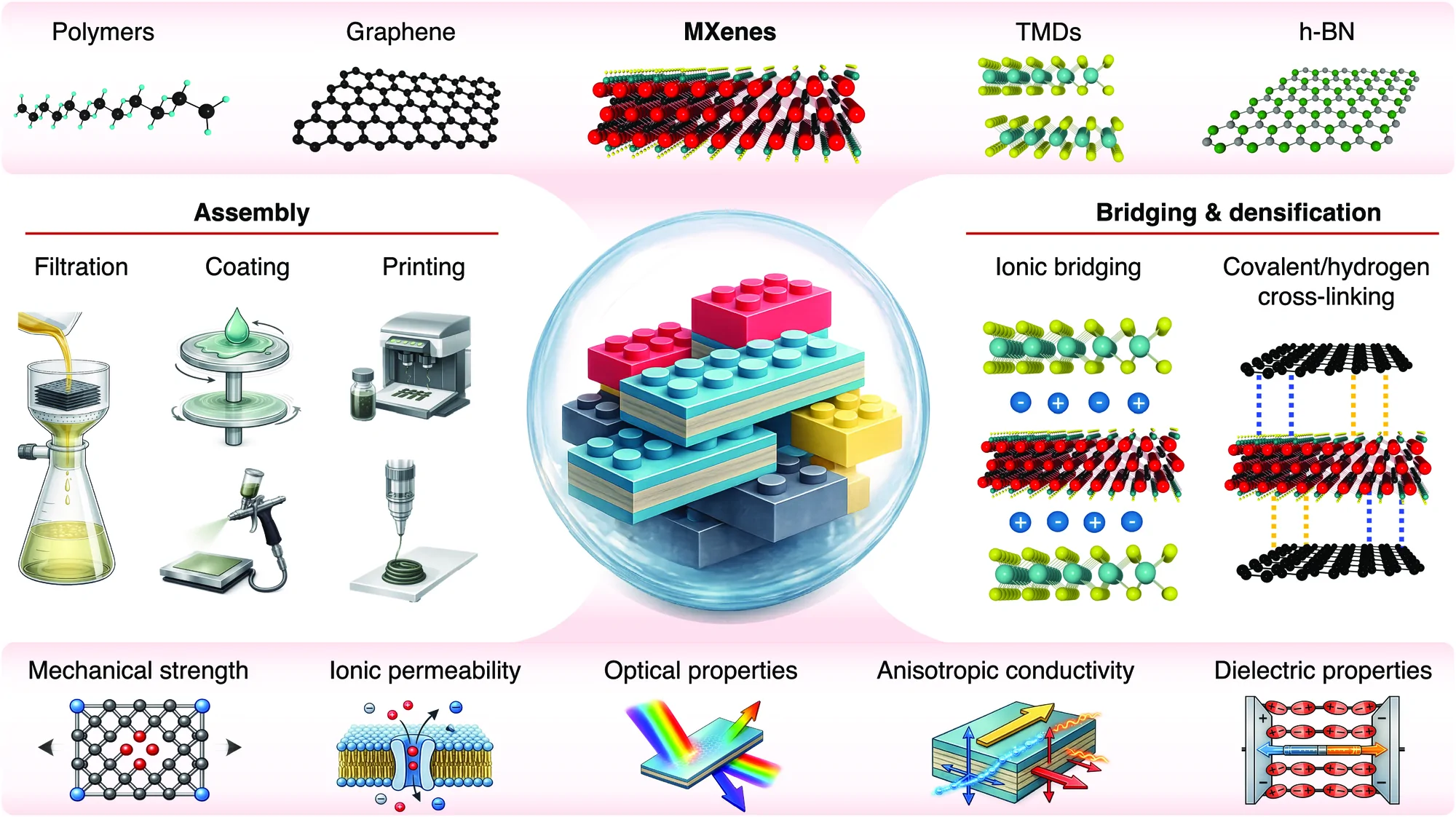
MXenes as nanoscale building blocks for assembled architectures and sustainable devices. Schematic illustration of MXenes as versatile components within a broader library of building blocks – polymers, graphene, transition-metal dichalcogenides (TMDs), and hexagonal boron nitride (h-BN) – that enable the design of layered and hybrid architectures. Through scalable assembly routes, including filtration, coating and printing, together with interfacial engineering by ionic bridging, cross-linking and densification, MXenes can be integrated with complementary 2D and soft-matter components across multiple length scales. Controlling these assembly strategies yields materials with tunable mechanical strength, ionic permeability, optical response, anisotropic electrical and thermal conductivity, as well as dielectric behavior, offering a modular pathway to multifunctional devices for water purification, energy storage, electromagnetic shielding, and related applications.

A clear example of this building-block concept is the recent progress in structural MXene films and laminates. Bridging-induced densification of Ti_3_C_2_T_*x*_ flakes through hydrogen bonding and covalent cross-links produces free-standing films with high tensile strength and conductivity, prepared entirely from dispersions in water.^125^ Later work combined Ti_3_C_2_T_*x*_ flakes with sequential organic and inorganic bridges to compact the films, pushing tensile strength into the gigapascal range without compromising toughness and electrical performance.^126^ Hybrid laminates where Ti_3_C_2_T_*x*_ is linked to neighboring graphene sheets can exceed 1 GPa in strength and offer ionotronic properties (high ionic and electronic conductivities) and redox capacitance, which shows that MXene flakes can lock otherwise loosely packed 2D fillers into dense and strong structures.^127^ More complex bridging schemes that use polymers or liquid metals have produced MXene films that can be rolled and cut for integration as structural and multifunctional elements in various devices.^128,129^ These studies treat MXenes not as passive fillers but as structural and functional building blocks in the architecture of assembled materials, transforming simple colloidal dispersions into components for structural batteries, actuators, and biointegrated electronics.

MXenes are equally powerful as building blocks in polymer and hybrid composites for thermal management and electromagnetic protection. High IR reflectivity, adjustable emissivity and strong interfacial interactions with polymers allow thin MXene layers to regulate heat flow within the films and coatings. For example, transparent ultrahigh-molecular-weight polyethylene (UHMWPE)/MXene films prepared by melt blending and hot pressing remain transparent in the visible range but absorb UV light and exhibit rapid photothermal heating, which has been translated into greenhouse covers that reduce annual cooling demand by tens of MJ m^–2^.^130^ Composites based on poly­(p-phenylene-2,6-benzobisoxazole) (PBO) and Ti_3_C_2_T_*x*_ combine high tensile strength with electrical insulation and in-plane thermal conductivity above 40 W m^–1^ K^–1^ in a water-processed film.^131^ Three-dimensional MXene/graphene polymer networks obtained through controlled filler segregation create anisotropic heat pathways at relatively low filler content so that conventional metal heat spreaders can be reduced or, in some cases, avoided.^132^ MXene-containing aramid nanofiber and “sweat-driven” elastomer composites further show that a single assembled material can act as mechanical support, electrical conductor, thermal regulator and energy-storage component in wearable systems.^133,134^ Many of these MXene-based composites are processed from dispersions or powders using industrially relevant routes such as melt blending, solution casting, filtration, hot pressing and simple coating steps.

Assembly concepts extend beyond flat films. Universal salt-assisted assembly deposits MXenes from aqueous suspensions onto chemically inert polymer substrates, producing MXene-functionalized plastics without the use of aggressive chemicals or organic solvents.^135^ MXene-coated 3D-printed nylon waveguides can carry Ku–Ka band signals used in low-Earth-orbit satellites with performance comparable to Al waveguides but at roughly one-eighth the weight, since only a thin, water-processed MXene layer replaces the bulky metal.^136^ Moreover, their manufacturing is much less expensive. These examples indicate that MXene flakes can be assembled onto lightweight and inexpensive supports to substitute heavy metallic components in electromagnetic interference shielding, antennas and wave-guiding hardware in a very wide range of frequencies, cutting material demand and decreasing fuel consumption in weight-sensitive sectors such as aerospace.

Looking ahead, we argue that MXenes can be used as programmable building blocks within data-guided assembly strategies, not only as isolated materials. Bibliometric and data mining studies have already highlighted the vast compositional and structural space for MXenes and their hybrids.^137^ Combining computational screening and machine learning with systematic experiments on assembly protocols shall make it possible to design architectures that meet performance targets and sustainability constraints simultaneously (e.g., greenhouse films with the required optical and thermal response that are straightforward to recycle). In parallel, the field would benefit from standardized protocols, especially for aging and recyclability, so that fair comparisons between MXene assemblies and industrial benchmarks can be performed. Coordinated efforts across these areas could make MXene building blocks the foundation for multifunctional, resource-efficient materials that support future sustainable infrastructure and devices.

## Outlook and Roadmap

MXenes are rapidly emerging as a versatile platform for environmental technologies as they combine multiple useful properties within a single material family. However, their environmental applications are not all at the same stage of technological maturity. In the near term, the most realistic opportunities are those that exploit properties already demonstrated in processable films, coatings, fibers, or powders. These include electromagnetic and multispectral shielding, passive thermal-management coatings, conductive additives and lightweight current collectors for batteries, dry bioelectronic electrodes, and selected chemical or strain sensors. These applications benefit from well-established MXene advantages, including high electrical conductivity at low thicknesses, water-based processing, and compatibility with existing coating, filtration, printing, and composite-manufacturing methods.

In the midterm, MXenes are well positioned for water purification, desalination, metal and rare-metal recovery, industrial wastewater treatment, capacitive deionization, smart windows, solar-driven evaporation, and salinity-gradient or “blue-energy” harvesting. Their tunable surface-termination chemistry, polar and charged surfaces, hydrophilicity, adjustable interlayer spacing, photothermal response, and mixed electronic/ionic transport enable MXene membranes and adsorbents to interact strongly with heavy metals, radionuclides, dyes, organic contaminants, and other pollutants, while their tunable interlayer galleries offer opportunities for molecular and ionic sieving. In parallel, MXene electrodes and membranes can support low-pressure separations, ion-selective transport, and electrochemically driven water-treatment processes powered by renewable electricity. Further progress will depend on validation under realistic operating conditions, including complex water chemistries, long-term operation, and scalable device configurations.

In the longer term, MXenes may play important roles in electrochemical environmental remediation, electrocatalytic conversion, distributed air-quality monitoring, healthcare devices, and MXene-enabled photovoltaic architectures. Their high electrical conductivity and electrocatalytic activity make them attractive electrodes for the electrochemical reduction of organic contaminants and for the electrocatalytic conversion of nitrates, CO_2_, or other pollutants into useful products. These electrochemical routes align with the broader electrification of environmental infrastructure, where treatment processes are powered by renewable energy rather than chemical reagents. MXene thin films are also highly sensitive to gases and humidity, suggesting low-power sensors for real-time monitoring of pollutants such as NO_2_, NH_3_, and volatile organic compounds. When integrated into distributed sensor networks, such devices could support responsive environmental management and early warning systems. MXene sensors and electrodes may also contribute to healthcare, particularly in wearable or bioelectronic devices, provided that stability, biocompatibility, calibration, and manufacturing reproducibility are established.

Across all maturity levels, sustainability should be treated as a full-system design constraint. Challenges in large-scale synthesis, oxidation and hydrolysis stability, recyclability, and end-of-life sustainability must be considered alongside device performance. Encouragingly, progress in greener etching chemistries, direct synthesis from abundant and inexpensive precursors, formulation of polymer-MXene composites, and encapsulation strategies is rapidly improving durability and scalability. Nevertheless, these advances must be evaluated using life-cycle and techno-economic metrics to support MXenes as environmentally superior alternatives. If stability, recovery, and circular processing are managed by design, MXenes could become cornerstone materials for circular environmental technologies, providing ultrathin thermal insulation, purifying water and air, recovering valuable resources, and enabling electrified, low-carbon remediation systems.

Finally, the rapidly advancing fields of data mining, high-throughput computation, and AI-assisted materials development are likely to dramatically expand the accessible space of MXene structures, compositions, and emergent properties. With thousands of theoretically predicted MXene variants, including ordered, disordered, high-entropy, vacancy-engineered, and heterostructured systems, the combinatorial design space far exceeds what can be explored experimentally by trial and error. Machine learning models trained on DFT data sets and experimental synthesis outcomes can accelerate the identification of stable compositions, low-energy etching pathways with reduced chemical waste, oxidation-resistant surface functionalization, and target functionalities such as optimized work function for photovoltaics, near-ideal adsorption energies for HER/OER or CO_2_ reduction, selective ion sieving for desalination, or tailored infrared emissivity for passive thermal management. Generative algorithms and inverse-design approaches may further propose entirely new MAX/MXene precursors based on abundance, cost, and environmental metrics, enabling design strategies aligned with sustainability goals. In this way, MXene research may evolve from empirical exploration toward predictive, sustainability-driven design, delivering purpose-built materials in a resource-constrained world.

## Data Availability

The survey data used and analyzed during the preparation of this Perspective are available from the corresponding authors on reasonable request.
